# From Pilot to Practice: A Large-Scale Evaluation of Gastroenterology E-Consultations at an Academic Medical Center

**DOI:** 10.1016/j.gastha.2026.100987

**Published:** 2026-04-30

**Authors:** Christian Bonilla, Sonali Palchaudhuri, Michael Thiim, Jason H. Wasfy, Susan A. Goldstein, Meera Changela, James M. Richter

**Affiliations:** 1 Division of Gastroenterology, Department of Medicine, Mass General Brigham, Harvard Medical School, Boston, Massachusetts; 2 Division of Cardiology, Department of Medicine, Heart and Vascular Institute, Mass General Brigham, Harvard Medical School, Boston, Massachusetts

**Keywords:** Electronic Consultation, E-Consult, Gastroenterology, Access to Specialty Care, Provider Satisfaction

## Abstract

**Background and Aims:**

Electronic consultations (e-consults) are asynchronous, provider-to-provider exchanges that allow specialists to provide clinical guidance without an in-person visit. Referring clinicians submit focused questions through the electronic health record, and specialists review relevant history and data to make recommendations. At many academic centers, waits for specialty appointments exceed months, limiting timely care and underscoring the need for alternative referral pathways. We evaluated the implementation, utilization, and provider experiences of a gastroenterology e-consult program at Massachusetts General Hospital.

**Methods:**

We analyzed all gastroenterology e-consults from January 2023 through September 2024, extracting completion rates, turnaround times, and clinical indications from 2416 encounters. Anonymous 5-point Likert surveys assessed satisfaction, perceived utility, and implementation barriers among referring providers and consulting gastroenterologists.

**Results:**

Of 2416 e-consults involving 2038 unique patients, 2112 (87%) were completed, with a mean turnaround time of 2.6 business days. Hepatology and abnormal imaging findings comprised nearly half of all requests. Referring providers reported high satisfaction and educational value, with most indicating that e-consults reduced unnecessary visits and testing. Consultants recognized clinical utility but cited inadequate reimbursement and difficulty meeting turnaround targets.

**Conclusion:**

Gastroenterology e-consults can operate effectively at scale, particularly for objective, data-driven questions such as abnormal liver function tests and imaging findings. However, the satisfaction gap between referring providers and consultants, driven largely by reimbursement and workload pressures, threatens long-term sustainability. Addressing these challenges through payment models that reflect cognitive work and streamlined workflows will be essential to maintain specialist engagement while expanding access to care.

## Introduction

Electronic consultations (e-consults) are asynchronous, provider-to-provider exchanges conducted through a shared electronic health record (EHR) or secure web platform.^1^ These structured interactions have gained prominence as health systems confront unprecedented demand for specialty care. A 2022 *Merritt Hawkins* survey found that across 15 major US cities, new patients waited an average of 26 days to see a physician in 5 key specialties.^2^ At Massachusetts General Hospital (MGH), a tertiary academic center within Mass General Brigham (MGB), the volume of clinic consult requests exceeds capacity, and the average scheduled wait time for an in-person gastroenterology appointment approaches 4 months, underscoring the need for alternative access pathways.

EHRs have transformed specialty consultations by enabling specialists to review patient data remotely, including laboratory results, imaging studies, and complete medical histories. This approach may allow for meaningful recommendations without requiring an in-person visit in certain scenarios.^3^, ^4^, ^5^ At MGH, e-consults began with a cardiology pilot program in 2014 and have since expanded to other specialties like gastroenterology, with early studies demonstrating high provider satisfaction and the ability to resolve many questions without in-person evaluation.^6^, ^7^, ^8^

Despite promising pilot results, we still lack comprehensive data on how e-consults perform at scale, evaluating usage patterns, provider satisfaction, and long-term sustainability. Key questions remain: Which clinical scenarios are best suited to e-consults? How do referring and consulting clinicians experience these programs? What operational challenges emerge when moving beyond the pilot phase?

This study analyzes over 2400 gastroenterology e-consultations across 21 months at MGH, examining clinical use patterns, consultation indications, and provider experiences. We surveyed both referring providers and consulting specialists to assess satisfaction, perceived clinical utility, and implementation barriers, providing insights into how e-consult programs function at scale in a large academic medical center.

## Materials and Methods

We analyzed all gastroenterology e-consult requests submitted at MGH from January 2023 through September 2024. The gastroenterology e-consult program operated through a shared enterprise-wide EHR (Epic), enabling access to patient data for both referring providers and consulting gastroenterologists within the MGB network. The gastroenterology e-consult program operated within an institutionally funded platform. Fifteen gastroenterologists served 545 authorized clinicians at the main hospital and 5 affiliated sites. Consultants received institutional compensation of approximately 1 relative value unit per e-consult, without dedicated protected time for review.

**Figure 1:**
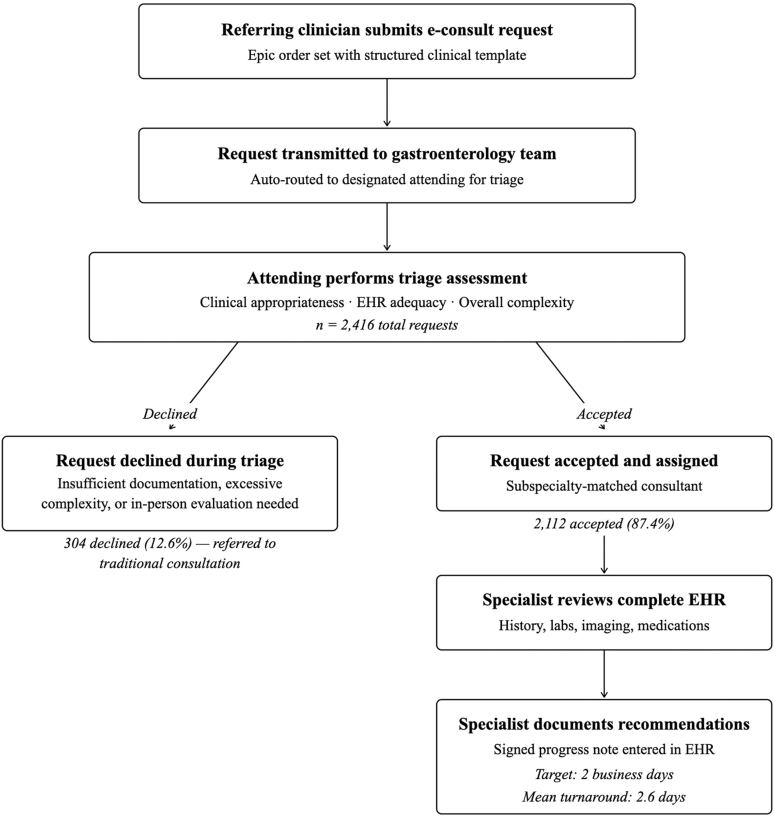
MGH gastroenterology e-consult workflow. Flow diagram of the e-consult process from submission through triage, subspecialty-matched consultant review, and entry of a signed progress note. Of 2416 requests, 2112 (87.4%) were accepted and 304 (12.6%) declined; mean turnaround was 2.6 business days against a 2-business-day target.

Referring clinicians accessed the service through a structured EHR order set that prompted a focused clinical question. Each submission underwent initial triage by a designated gastroenterologist who applied predefined criteria: (1) clinical appropriateness for asynchronous management, (2) adequacy of documentation in the EHR, and (3) overall complexity of the patient and clinical question. Requests deemed inappropriate for electronic consultation were declined (due to need for in-person evaluation, insufficient clinical documentation, or excessive complexity), and the referring provider was advised to submit a traditional consultation request. Accepted cases were routed to a subspecialty-matched consultant who reviewed the full EHR and entered recommendations as a signed progress note within a 2-business-day turnaround target (Figure 1).

Using a standardized EHR extraction protocol, we reviewed all 2416 e-consult requests submitted during the study period. For each request, we captured the submission and completion timestamps, triage disposition, consulting and referring clinician identifiers, practice location, and patient medical record number. Operational metrics included total number of e-consults, completion rates, response times (measured in business days from initial submission to consultant response), number of unique patients served, and participating clinician counts. Completed e-consults were defined as those resulting in specialist recommendations, while incomplete consultations included those declined during triage or canceled by referring providers.

Each e-consult was categorized according to its primary clinical indication using diagnostic categories developed by the gastroenterology team. A reviewer assigned clinical categories based on gastroenterology subspecialty areas and clinical presentation patterns. Geographic analysis examined the distribution of e-consults across hospital-based practices and network-affiliated sites.

To characterize user experience, we administered parallel anonymous surveys. Survey instruments were adapted from previously published e-consult evaluations and used 5-point Likert scales (1 = strongly disagree, 5 = strongly agree) to evaluate perceptions of e-consult usefulness, efficiency, patient care impact, satisfaction, and future use intent. The referring-clinician questionnaire assessed clarity of recommendations, educational value, perceived impact on patient care, avoidance of visits or tests, timeliness, overall satisfaction, and intent to continue use. The consultant questionnaire assessed perceived usefulness, communication facilitation, feasibility of meeting the 2-day target, adequacy of reimbursement, overall satisfaction, and intent to remain a program participant.

Survey links were sent to clinicians who had submitted or completed at least 1 e-consult during the final study quarter (200 referrers and all 15 consultants). Response rates were 27% (53/200) for referrers and 53% (8/15) for consultants.

## Results

**Figure 2:**
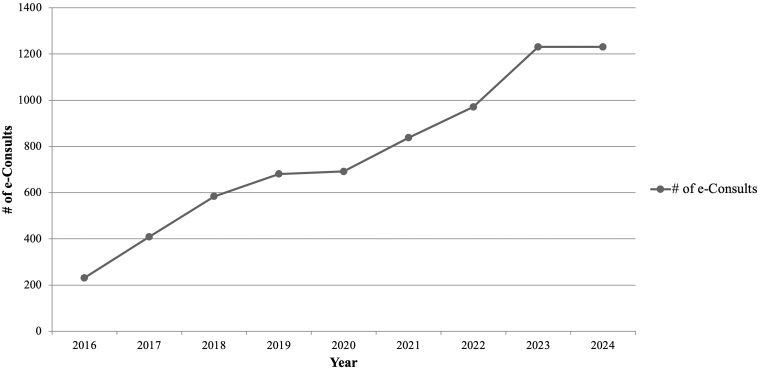
MGH e-consult order volume by year. Annual gastroenterology e-consult volume at Massachusetts General Hospital, 2016–2024.

Between January 2023 and September 2024, a total of 2416 gastroenterology e-consult requests involving 2038 unique patients were submitted across the MGB network. Of these, 2112 (87%) were completed by 15 gastroenterologists, and 304 (13%) were declined during triage. The mean turnaround time from submission to specialist recommendation was 2.6 business days. Requests were placed by 545 clinicians; 1604 of 2112 completed e-consults (76.0%) originated from MGH providers, and 508 (24.0%) from affiliated institutions, including MGB Community Physicians, North Shore Hospital affiliates, Martha’s Vineyard Hospital, Brigham and Women’s Hospital, and Newton-Wellesley Hospital. Since the program’s inception in 2016, annual volume has increased from approximately 200 consultations per year to over 1200 per year by 2024 (Figure 2).

Primary clinical indications are shown in Table. Hepatology-related issues were most common, accounting for 569 of 2112 completed e-consults (26.9%), largely evaluations of abnormal liver function tests (473 of 569). Abnormal imaging findings accounted for 464 e-consults (22.0%). Together, hepatology and imaging comprised nearly half of all consultations (1033 of 2112). Symptom-based referrals totaled 357 (16.9%), most often diarrhea (135 cases), abdominal pain (59 cases), and dyspepsia (50 cases). Inflammatory or autoimmune conditions accounted for 174 (8.2%), and oncology or screening questions for 150 (7.1%). The remaining 398 consultations (18.8%) involved surveillance, pancreatic or biliary disorders, and miscellaneous indications.

**Table:** Primary Clinical Domains of Completed Gastroenterology E-Consults and Representative Examples, MGH (January 2023 to September 2024; n = 2112)

Clinical domain	No. (%)	Examples (no.)
Hepatology	569 (26.9)	Abnormal liver function tests (473), fatty liver disease (40), hepatitis (23), elevated ferritin (23), cirrhosis (6), abnormal copper levels (4)
Abnormal imaging findings	464 (22.0)	Liver, pancreatic, or biliary abnormalities detected on imaging (464)
Gastrointestinal symptoms	357 (16.9)	Diarrhea (135), abdominal pain (59), dyspepsia (50), constipation (25), bloating (23), nausea/vomiting (21), GERD (14), irritable bowel syndrome (16), motility disorders (14)
Inflammatory/autoimmune conditions	174 (8.2)	Autoimmune markers (105), celiac disease (29), diverticular disease (14), *Helicobacter pylori* (10), peptic ulcer disease (8), IBD (4), eosinophilic esophagitis (4)
Oncology and screening	150 (7.1)	Colon cancer related (67), colonic polyps (40), GI cancer screening (21), Barrett’s esophagus (16), FIT/FOBT follow-up (6)
Surveillance/follow-up	107 (5.1)	General surveillance/follow-up (101), endoscopy follow-up (6)
Pancreatic/biliary disorders	45 (2.1)	Pancreatic disorders (33), elevated lipase (8), gallbladder/biliary disease (4)
Other	246 (11.6)	Anemia (40), GI bleeding (14), medication review (10), weight loss (10), hemorrhoids (10), miscellaneous GI conditions (162)

FIT, fecal immunochemical test; FOBT, fecal occult blood test; GERD, gastroesophageal reflux disease; IBD, inflammatory bowel disease.

**Figure 3:**
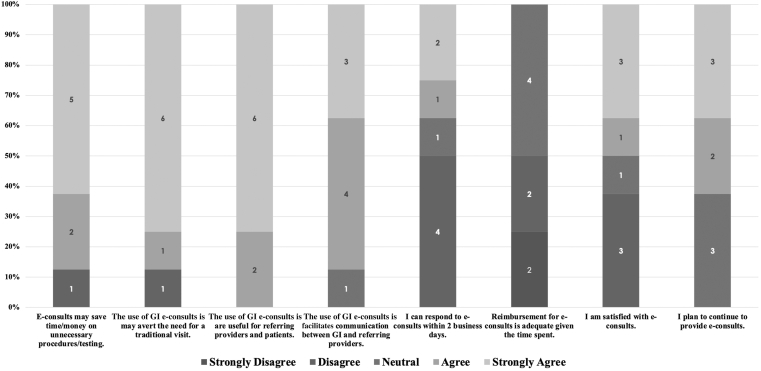
Consulting gastroenterologists' ratings of the MGH gastroenterology e-consult program (5-point Likert scale; n = 8). Stacked bar chart showing the distribution of consultant responses across program domains.

Eight of the 15 gastroenterologists completed the consultant survey (Figure 3). The majority agreed that e-consults reduce unnecessary procedures and testing (88%) and may avert the need for traditional visits (88%). All respondents found e-consults useful for referring providers and patients (100%), and 88% agreed they facilitate communication between gastroenterology and referring providers. Opinions on the feasibility of responding within 2 business days were more divided, with 38% agreeing and 50% disagreeing. Reimbursement was viewed as inadequate by all respondents; none agreed it was sufficient, with 50% actively disagreeing. Overall satisfaction was mixed (50% agreed, 38% disagreed), though 63% indicated plans to continue providing e-consults.

**Figure 4:**
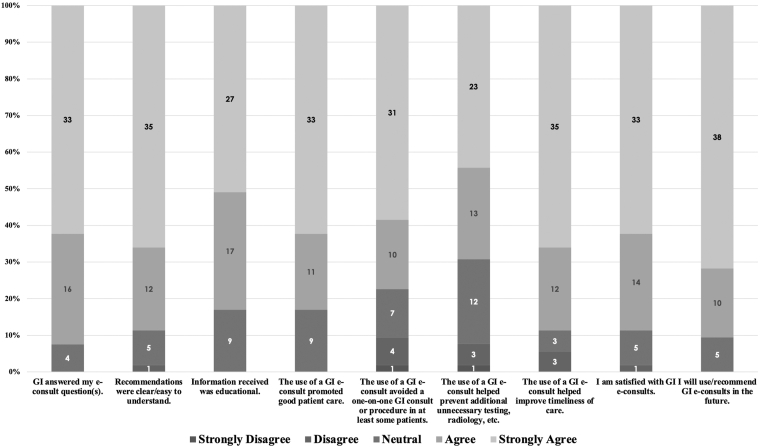
Referring clinicians’ ratings of the MGH gastroenterology e-consult program (5-point Likert scale; n = 53). Stacked bar chart showing the distribution of referring-clinician responses across program domains.

Among referring providers, 53 of 200 eligible clinicians completed the survey (Figure 4). Most reported that e-consults answered their clinical questions (62% strongly agreed, 30% agreed, 8% neutral) and that recommendations were clear and easy to understand (66% strongly agreed, 23% agreed, 9% neutral, 2% disagreed). The majority found the information educational (51% strongly agreed, 32% agreed, 17% neutral) and believed e-consults promoted good patient care (62% strongly agreed, 21% agreed, 17% neutral). Many reported that e-consults avoided at least some in-person consultations or procedures (58% strongly agreed, 19% agreed, 13% neutral, 8% disagreed, 2% strongly disagreed) and prevented unnecessary testing or radiology (43% strongly agreed, 25% agreed, 23% neutral, 6% disagreed, 2% strongly disagreed). Nearly 9 in 10 respondents felt e-consults improved timeliness of care (66% strongly agreed, 23% agreed, 6% neutral, 6% disagreed). Overall satisfaction was high (62% strongly agreed, 26% agreed, 9% neutral, 2% disagreed), and most indicated they would use or recommend e-consults in the future (72% strongly agreed, 19% agreed, 9% neutral).

## Discussion

In this large-scale evaluation of 2416 gastroenterology e-consults, we demonstrate the successful transition from pilot program to sustained clinical practice, identifying specific scenarios where e-consults are most effective and operational challenges that must be addressed for long-term sustainability.

Our findings reveal clinical scenarios particularly amenable to electronic consultation. Nearly half of consultations addressed abnormal liver function tests and imaging findings, reflecting the suitability of e-consultations for cases centered on objective data interpretation. These consultations typically involve reviewing laboratory values or radiographic findings that do not require physical examination. For imaging abnormalities and colorectal screening guidance, e-consultations provided an efficient mechanism for delivering guideline-concordant surveillance recommendations.

The potential for systematic improvement through electronic support tools merits consideration. Embedding decision tools within the EHR could encourage asymptomatic laboratory abnormalities or incidental imaging findings to e-consultation while directing symptomatic or high-acuity presentations to in-person care. Standardized protocols could further enhance efficiency. A hepatology pathway, for instance, could mandate specific laboratory studies, complete medication reconciliation, and relevant imaging before submission, thereby improving consultation quality. Outpatient care models in other specialties illustrate this approach. In venous thromboembolism, the HESTIA criteria and the simplified Pulmonary Embolism Severity Index have been prospectively validated to identify patients at low risk for short-term complications who can be treated at home with structured follow-up without increased rates of adverse events.^9^^,^^10^ Both models apply explicit, evidence-based criteria at the point of entry to direct patients to the least intensive setting consistent with safety. An example of embedding analogous rule-based triage into gastroenterology e-consults is to consider automated Fibrosis-4 scoring for hepatology referrals and mandatory inclusion of recent liver chemistries, complete blood counts, relevant imaging, and prior endoscopy reports. This could improve consultation quality by increasing the likelihood that referring providers submit the labs and imaging necessary for consultants to formulate a timely response.

The satisfaction differential between referring providers and consultants reveals a sustainability challenge. This gap likely reflects reimbursement structures that undervalue cognitive services relative to their time requirements. Medicare has historically compensated procedural care at rates several-fold higher per hour than evaluation and management services.^11^ While our study focused primarily on consultant experiences, e-consult programs also affect referring provider workload. In a qualitative study of safety-net primary care providers using a mandatory e-consult system, primary care providers consistently perceived e-consults as shifting specialty care work to them, with some viewing this as an acceptable trade-off for improved timeliness while others expressed frustration with increased administrative burden.^12^ The opt-in design of our program, which allows referring providers to choose between e-consult and traditional referral, may mitigate workload concerns compared to mandatory e-consult systems. Consultants identified reimbursement and turnaround time as areas of concern, findings consistent with a scoping review of 130 e-consult implementation studies identifying inadequate compensation and increased workload as primary barriers to specialist participation.^13^

Beyond immediate clinical care, many referring clinicians reported that e-consults had educational value, a finding consistent with prior work in which over 80% of primary care physicians valued the educational aspects of e-consultation systems.^14^ Future research should assess whether regular e-consult users demonstrate improved diagnostic completeness or altered referral patterns over time. While our study did not directly survey patients, existing literature suggests generally favorable patient experiences with e-consults. In a large multisite survey of over 8000 patients across 9 academic medical centers, patients who received e-consults reported similar satisfaction levels to those receiving traditional in-person referrals, with 78% preferring e-consults for similar problems in the future.^15^ Further research should directly assess patient satisfaction with gastroenterology e-consults.

Recent analyses show that well-designed e-consult programs can lower spending and improve access to specialty care. In the Veterans Health Administration, patients managed through e-consults had lower adjusted total costs at 3 and 6 months than those referred for in-person specialty visits, with most savings in outpatient care.^16^ In New York City Health + Hospitals, the nation’s largest safety net system, system-wide e-consult implementation was associated with higher scheduling of specialty referrals and shorter waits for appointments.^17^ At the University of Colorado School of Medicine, an academic medical center’s e-consult program eliminated more than 300,000 miles of patient travel and approximately US$57,000 in fuel costs.^18^ In our study, many gastroenterology referrals involved review of existing laboratory or imaging results. Managing these asynchronously may allow hospitals to reserve clinic time for patients requiring examination or procedures while reducing patient travel burden. A logical next step is to evaluate whether scaling gastroenterology e-consults shortens time to indicated procedures for patients requiring in-person care while maintaining or lowering overall costs.

As e-consult programs expand, managing specialist workload becomes critical. Our nearly 90% completion rate demonstrates feasibility, yet sustained growth will hinge on absorbing rising volumes without overburdening consultants. Emerging evidence suggests that artificial intelligence (AI)-based automation, such as GPT-enabled inbox assistants and AI ambient scribe systems, can reduce documentation time and cognitive load. In a study by Garcia et al, a GPT-based inbox assistant piloted with 162 clinicians significantly reduced perceived task load and work exhaustion.^19^ Separately, Duggan et al reported that the use of an AI ambient-scribe system for clinical documentation was associated with a 20% reduction in note-writing time and a 30% reduction in after-hours documentation time.^20^ Incorporating similar AI assistance to collate the relevant data to review into gastroenterology e-consult platforms may help expand access without proportionally increasing time burdens on specialists.

Ultimately, the sustainability of e-consults depends on aligning incentives, workflows, and quality metrics. Payment models must reflect the actual cognitive work of reviewing complex cases and generating evidence-based recommendations. Workflow designs should balance timeliness with feasibility, and quality frameworks should incorporate indicators such as diagnostic concordance, triage appropriateness, and recommendation implementation rates. Addressing these operational foundations will be essential for realizing the full potential of e-consults to improve access and value in gastroenterology care.

### Limitations

This study has important limitations. First, survey response rates were modest (27% for referring providers and 53% for consultants), which may introduce selection bias and either overestimate or underestimate satisfaction in both groups. Second, we lacked data on downstream outcomes, including whether e-consult recommendations were implemented, whether perceived reductions in in-person gastroenterology clinic and testing were realized, and the frequency of iterative communication between referring providers and consultants. We also did not analyze the distribution of e-consults across individual subspecialists, assess whether e-consult participation improved the quality of previsit workup for patients ultimately seen in person, or directly assess patient satisfaction. Third, as a single-institution study within 1 integrated health system, findings may not generalize to settings with different EHRs, payment structures, or patient populations. Finally, the 21-month observation period may not capture long-term sustainability challenges that could emerge as consultation volumes grow and initial program enthusiasm diminishes.

## Conclusion

In this large-scale evaluation of 2416 gastroenterology e-consults, we found high completion rates and rapid turnaround times, with expanded access across academic and community sites. However, the divergent satisfaction levels between referring clinicians and consulting specialists, driven largely by concerns about reimbursement and time pressures, raise critical questions about long-term sustainability. Our findings suggest that e-consults are particularly well suited for objective, data-driven questions such as abnormal liver function tests and imaging findings, providing both timely clinical guidance and educational value to referring providers. The successful implementation of e-consultation programs requires more than technological infrastructure. Health systems must develop payment models that appropriately compensate specialists for asynchronous clinical review, establish efficient workflows that reduce administrative burden, and implement systematic monitoring that identifies inequities in access to specialty care.
